# Studying Spatial Visual Attention: The Attention-Window Task as a Measurement Tool for the Shape and Maximum Spread of the Attention Window

**DOI:** 10.3389/fpsyg.2021.614077

**Published:** 2021-02-25

**Authors:** Stefanie Klatt, Daniel Memmert

**Affiliations:** ^1^Institute of Sports Science, University of Rostock, Rostock, Germany; ^2^Institute of Exercise Training and Sport Informatics, German Sport University Cologne, Cologne, Germany

**Keywords:** stimulus-onset asynchrony, predictive cues, peripheral cues, zoom lens model, attentional focus

## Abstract

Visual attentional processes have been an important topic in psychological research for years. Over the last few decades, new methods have been developed, aiming to explore the characteristics of the focus of attention in more detail. Studies that applied the “Attention-Window Task” (AWT) quantified the maximum extent of the “Attention Window” (AW) along its horizontal, vertical, and diagonal meridians, when subjects were required to perceive two peripheral stimuli simultaneously. In three experiments using the AWT, we investigated the effects of cue validity (Experiment 1), stimulus-onset asynchrony (SOA) (i.e., the interval between the onset of the cues and the onset of the target stimuli), and target stimuli complexity (Experiment 3) on the size and shape of the AW. Results showed that the AW was greater under valid cue conditions compared to invalid conditions, when the locations of cue and target stimuli differed. Furthermore, the AW decreased when the SOA between the cue and targets was reduced and also when the task complexity was higher and more objects within the target stimuli had to be classified. Overall, it can be stated that the AWT with its possible task changes and adjustments can be considered as a potential standard tool to measure the maximum spread and shape of the spatial AW.

## Introduction

Over the past few decades, psychologists have developed a series of methods to study visual attentional processes, in order to draw conclusions about the characteristics of the visual focus of attention (Carrasco, 2011). This focus is usually described as the area of the visual field where multiple stimuli can be identified simultaneously (Hüttermann et al., 2019b). A number of studies have demonstrated that perceptual capabilities are spread over a wider peripheral area than attentional capabilities (Klatt et al., 2020). Prominent metaphors turn the focus of attention to act like a spotlight (Posner, 1980), a zoom lens (Eriksen and St. James, 1986), or a gradient (LaBerge and Brown, 1989). According to the spotlight model, attention operates like a beam or a moving spotlight; i.e., stimuli on which the spotlight is focused are selected for priority processing at the expense of stimuli being presented outside. The zoom lens model of visual attention–an extension of the spotlight model–proposes that the size of the attentional focus can be adjusted and predicts a tradeoff between the size and processing efficiency because of limited processing capacities, much like a camera lens. When attentional resources are focused in a small area, the concentration of these resources leads to a greater facilitation of perceptual performance in comparison to when these resources are spread more thinly over a larger area. This means that with an increasing spatial spread of a unitary focus, processing efficacy, within the area that is attended, decreases. To complement this, gradient theories (e.g., LaBerge and Brown, 1989) infer that attention is organized in a gradient fashion around the attentional focus location–i.e., decreasing as the distance from the focus of attention increases. While all these models (spotlight, zoom lens, and gradient) assume a focus of attention that is not divisible, an opposing view postulates that attention can be divided between multiple objects (split attentional foci), suggesting that it is possible to focus attention on two non-contiguous areas (e.g., Castiello and Umiltà, 1992; Awh and Pashler, 2000). All the different models and approaches indicate that the distribution of visual attentional resources has not been fully explained yet.

An exemplary tool, which has been validated for measuring the maximum spread of attention and has been used increasingly in scientific research over the last few years, is the “Attention-Window Task” (AWT) (Hüttermann et al., 2013). The AWT determines the ability of an individual to disseminate visual attention peripherally when two sets of stimuli are presented simultaneously. The stimuli normally appear equidistant from the center of the projection screen at two locations along the focus’ horizontal, vertical, or one of its two diagonal meridians within milliseconds. The maximum extent of the focus of attention is commonly defined as the greatest separation between the target stimuli along each meridian, until the point where participants can still identify both stimuli correctly with a minimum response rate of 75% (Hüttermann and Memmert, 2017). Researchers have previously determined an “Attention Window” (AW) (which equals the maximum spread of attention) by drawing a line between the measured “endpoints” (maximum target stimuli separations) of the focus’ horizontal, vertical, and diagonal meridians (cf. Hüttermann and Memmert, 2014, 2015; Hüttermann et al., 2014). This approach uncovered a clearly defined spatial area for the maximum AW in which two peripheral stimuli can be perceived at the same time.

The AWT has been applied in a number of studies investigating the impact of various factors on the shape and size of the AW; for instance, physical exercise (e.g., Hüttermann and Memmert, 2014), emotional and motivational states (e.g., Hüttermann and Memmert, 2015), body/head position (e.g., Hüttermann et al., 2019b), presentation surface (e.g., Klatt and Smeeton, 2019), creativity (e.g., Hüttermann et al., 2019a), decision making (e.g., Hüttermann et al., 2018), sporting expertise (e.g., Hüttermann et al., 2014; Hüttermann and Memmert, 2018), and age (e.g., Hüttermann et al., 2012). Brocher et al. (2018) showed a link between task effort and pupil size, which can be used to track the degree to which people covertly extend their AW or detect stimuli in their peripheral vision. The evaluation of stimuli farther away from eye fixation requires more effort than the evaluation of stimuli closer to eye fixation. The authors of that study proved the AWT to be a powerful tool to assess people’s maximum AW.

With reference to current literature, three primary findings can be cited regarding the previously investigated characteristics of the AW (for a review article, see Hüttermann and Memmert, 2017). First, the AW is about five to six times smaller than the visual field. Second, processing accuracy decreases with increasing eccentricity, meaning the closer the peripheral target stimuli are to the threshold of the AW, the more difficult it is to perceive them. Third, the maximum AW presents itself in the shape of an ellipse with a wider separation between horizontally presented stimuli than vertically presented ones. Reliability and validity of the AWT measures have been tested in previous research; the authors found medium–high test–retest reliabilities (0.8) (e.g., Kreitz et al., 2015a, b).

Earlier research determining the AW so far has always used the same basic AWT and has investigated some effects on its size and shape by manipulating the subjects’ physical load, head position, emotional or motivational states, etc. (for an overview, see Hüttermann and Memmert, 2017). The current study investigated, for the first time, the effects of task-specific changes on the size and shape of the AW by adjustments and modifications to the basic task in order to understand the mechanisms fundamental to the processing of peripheral stimuli even better. Three experiments were conducted with slightly modified versions of the basic AWT; specifically, we examined the effects of cue validity (Experiment 1), stimulus-onset asynchrony (SOA) (i.e., the interval between the onset of the cues and the onset of the target stimuli), and target stimuli complexity (Experiment 3) on the maximum spread and shape of the AW.

## Experiment 1

Experiment 1 was aimed at investigating whether a variation in the cue stimuli has an influence on the performance in the AWT. Until now, solely peripheral, valid cue stimuli have been used in the AWT in previous studies. In order to understand and analyze the effects of any pre-cues in more detail, it was considered necessary to differentiate between peripheral cues, both valid and invalid cues. In general, cue stimuli provide information about the location of the presentation of the subsequent target stimuli. When a cue correctly indicates the target location, it is considered a valid cue; otherwise, it is invalid. An invalid condition involves presentation of a cue that provides incorrectly predictive information about the position of the subsequent target stimulus–because cue and target stimulus are presented at some other locations (Riggio and Kirsner, 1997). The current research followed in the footsteps of previous perception or attentional research that has made use of validly and invalidly cueing objects (e.g., Posner and Cohen, 1984).

There is evidence that valid peripheral cues drive rapid and automatic facilitation of target detection (e.g., Posner and Cohen, 1984; Maylor, 1985; Possamai, 1986). Valid cue stimuli, as opposed to invalid cue stimuli, enable the initiation and, possibly also, the finalization of the process of position selection before the presentation of the target cues (Müller and Rabbitt, 1989). However, most previous research has applied methods using one single peripheral cue leading to the situation where subjects primarily narrowed their attention around this cue (Yeshurun, 2019).

In contrast to these studies, the current one was related to the spatial spread of attention, i.e., the subjects were required to extend their attention in order to perceive peripheral target stimuli along the largest possible AW. They performed both the basic version of the AWT with valid cue stimuli and a modified version with a combination of valid and invalid cue stimuli. Following the results of previous research using peripheral cues–even with a single peripheral stimulus (cf. Deubel, 2008; Moehler and Fiehler, 2018)–we expected to find slightly decreasing general attentional performance (operationalized by the size/the spread of the AW) in the modified AWT. We also expected worse attentional performances for only these trials including valid (predictive) cues in the modified AWT as participants did not know whether the peripheral cues were valid or invalid (non-predictive) compared to the basic AWT (with solely valid peripheral cues). Furthermore, we expected to find decreasing attentional performance in invalid trials compared to valid ones.

### Participants

To calculate sample size requirements, G^∗^Power 3.1 (Faul et al., 2009) was used. Power analysis indicated that a sample size of at least 15 participants would result in a power of 0.8 (α = 0.05, *f* = 0.25). In order to adjust for any potential technical problems or data recording, we decided to employ 20 subjects in advance for each experiment.

A total of 20 participants (8 female and 12 male participants) aged 19 to 31 years [mean_*age*_ = 22.35 years, standard deviation (SD) = 2.81 years] took part in Experiment 1. All participants reported normal or corrected-to-normal (with contact lenses) vision. The experiment was carried out in accordance with the Helsinki Declaration of 1975, and written informed consent was obtained from each participant prior to testing. Approval was obtained from the lead institution’s ethics board.

### Materials and Procedure

**FIGURE 1:**
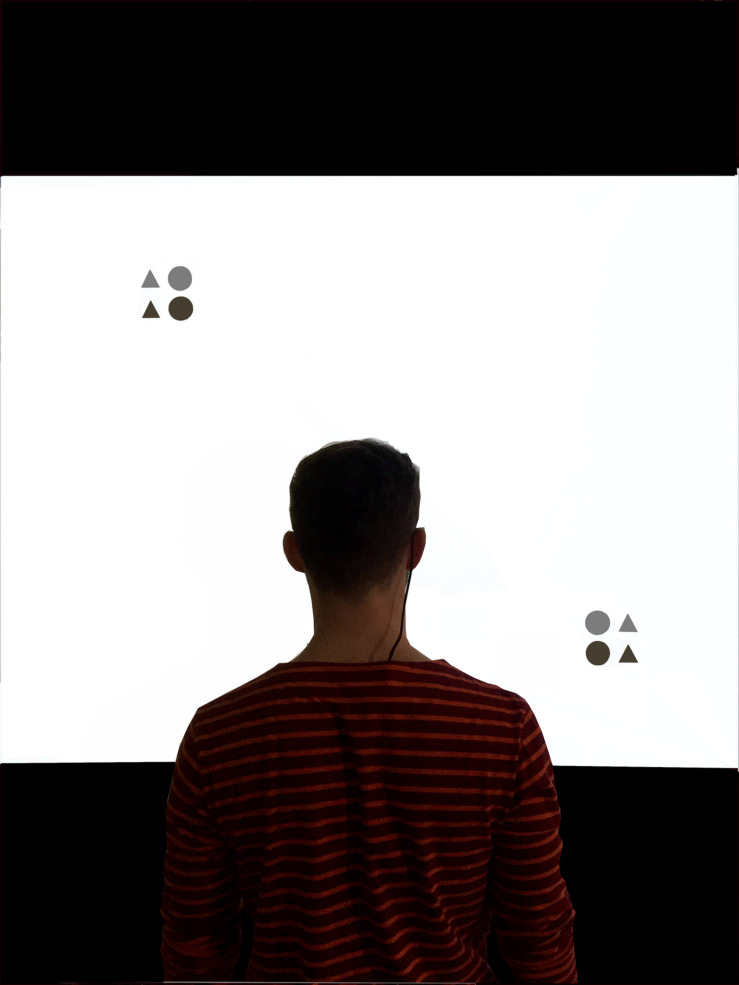
The experimental setup with a participant wearing the mobile eye-tracking system, standing in front of the screen while completing the Attention-Window Task with target stimuli presented along the diagonal meridian.

Participants were tested individually in a laboratory room. They stood approximately 1.30 m from a 2.80 × 2.20-m white projection screen (Figure 1). Every participant performed both the basic version of the AWT with valid cue stimuli and a version of modified AWT with a combination of valid and invalid cue stimuli. Participants performed the different versions of the task in a random order. Before the completion of each AWT version, participants performed 12 additional practice trials.

Participants were required to focus their gaze at the central fixation cross throughout each trial in the AWT. Fixation of the gaze was monitored with a mobile video-based eye tracking system (SMI eye tracking glasses, 30-Hz recording; SensoMotoric Instruments). Depending on the task version (basic or modified AWT), they were informed that pre-cues would give either valid or invalid information about the position of the target cues. The implementation of every version of the AWT with 288 trials each took approximately 10 to 15 min.

#### Basic AWT

The original AWT (basic task) developed by Hüttermann et al. (2013) was presented using E-Prime 2.0 (Psychology Software Tools, Pittsburgh, PA, United States). Different stimulus pairs were displayed along one of the four meridians (one horizontal, one vertical, and two diagonal) separated by 45° rotation at equal distance from the screen center. The distance varied randomly between 10° and 45° of visual angle, in increments of 5° of visual angle for the total number of 288 trials in each version (basic and modified) of the AWT. A 30-s break followed after every 72 trials.

**FIGURE 2:**
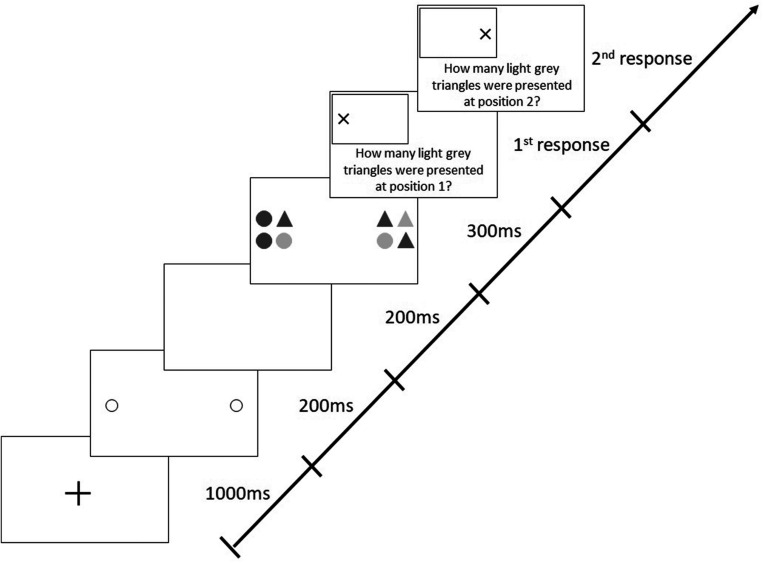
Sequence of events in a trial with target stimuli presentation along the horizontal meridian following valid (predictive), peripheral cues in the Attention-Window Task (modified from Hüttermann et al., 2013).

Each trial began with a central, black fixation cross (1,000 ms), followed by two 200-ms pre-cue circles indicating the future locations of the two target stimuli (Figure 2). After a 200-ms blank interval, the stimuli appeared for 300 ms. Each stimulus (19 × 19 cm) comprised four elements (9 × 9 cm; with a gap of 1 cm between the elements), which were circles or triangles filled in light or dark gray. This means that there were four different possible elements in total. The form (circle and triangle) and shading (light gray and dark gray) of each of the elements within one stimulus varied randomly from trial to trial. Participants had to identify the number of light gray triangles in each stimulus and were required to give their responses verbally. The probability of presenting zero, one, two, three, or four light gray triangles in a stimulus was 20% each. Participants were required to differentiate between both the color and the shape of stimuli (i.e., triangle and light gray), therefore demanding visual attention (Hüttermann et al., 2019c).

#### Modified Version of the AWT

**FIGURE 3:**
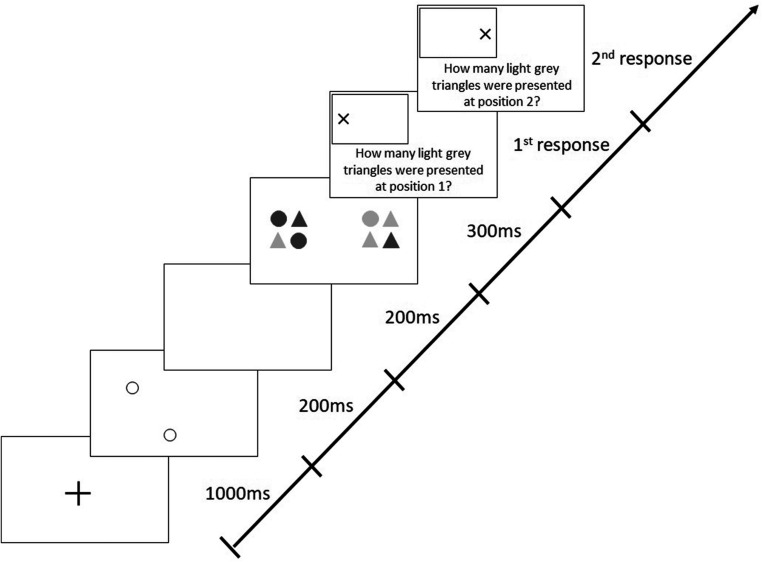
Sequence of events in a trial with target stimuli presentation along the horizontal meridian following invalid (non-predictive), peripheral cues in the modified Attention-Window Task (modified from Hüttermann et al., 2013).

The version of the AWT, which included 288 trials with a mix of valid and invalid cues, differed from the original, basic task in which valid cues were presented in 80% of trials and invalid cues in the remaining 20%. In valid trials, cues were presented at the positions where the target stimuli appeared later on (like in all trials in the basic AWT, cf. Figure 2), whereas in invalid trials, positions of cues and target stimuli were different; i.e., invalid cues could appear along any of the three non-target meridians at randomly selected different positions (Figure 3).

### Data Analysis

Responses were registered as correct only for trials where participants reported the right number of light gray triangles at both stimulus locations. Starting at the smallest distance between the two peripheral stimuli, a threshold was ascertained until which participants could correctly identify both stimuli with a probability of at least 75%. This procedure has also been used in previous research applying the AWT and defining the AW as the largest stimulus separation where each participant reliably identified the number of light gray triangles in both target stimuli in at least 75% of the trials (cf. Hüttermann et al., 2014). The measurement started from the smallest stimulus separation, i.e., 10°, continuing to the next greater visual angle, i.e., 15°, and so on. As soon as the accuracy was less than 75% at one visual angle (e.g., at 30°), the closest smaller stimulus separation (in this example, 25°) was determined as the subject’s maximum expansion of his/her visual attention. This procedure was applied for every single meridian, and finally, a maximum AW was determined by drawing a line between the measured endpoints of the different meridians in order to mark out the greatest possible accessible surface in which two peripheral stimuli can be perceived simultaneously (Hüttermann and Memmert, 2014, 2015). This approach uncovered a clearly defined spatial area, the maximum AW. Subsequently, we compared the separations corresponding to the 75% accuracy threshold of the AW in a 2 × 3 [AWT version (basic task, modified task) × meridian (horizontal, vertical, diagonal)^1^ ] repeated-measures analysis of variance (ANOVA). This ANOVA was performed in order to examine the differences of the maximum extension of the AW between the two different task versions (basic AWT, modified AWT). We also performed two additional 2 × 3 ANOVAs to analyze the effects of the different trial conditions within the tasks in more detail. Keeping the dependent variable the same, we conducted one ANOVA with trial condition (valid trials of the basic task and valid trials of the modified task) and meridian as repeated-measures within-subjects factors and another ANOVA with only one difference, namely, that the factor condition included the valid and the invalid trials of the modified task. For analyses in which the sphericity assumption was violated, we reported the value of ε from the Greenhouse–Geisser correction.

### Results and Discussion

**FIGURE 4:**
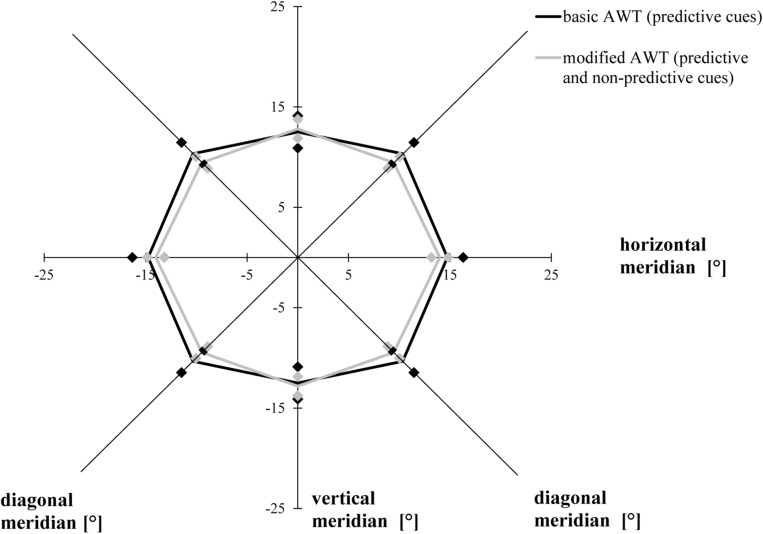
Attention Window with 75% correct performance as a function of meridian and task condition (basic task and modified task including predictive and non-predictive trials). Error bars (indicated by diamond/square symbols) indicate the 95% confidence interval.

The ANOVA with the factor task condition (basic AWT, modified AWT including all trials) revealed similar AW sizes for the two tasks, *F*(1, 19) = 0.455, *p* = 0.508, η^2^ = 0.023. There was a primary effect for the factor meridian, *F*(1.542, 29.293) = 18.835, *p* < 0.001, η*_*p*_*^2^ = 0.498, ε = 0.771 (Mauchly’s test of sphericity: χ^2^(2) = 6.350, *p* = 0.042): Bonferroni-corrected follow-up pairwise comparisons showed a wider alignment of the participants’ AW along the horizontal meridian compared to the vertical meridian (*p* < 0.001) or the diagonal meridian (*p* < 0.001), with no difference between the diagonal and the vertical meridians (*p* = 0.841). This indicated an elliptical shape of the AW. The interaction between condition and meridian was significant, *F*(2, 38) = 4.020, *p* = 0.026, η^2^ = 0.175. However, there was no significant difference between both tasks for the horizontal (*p* = 0.426) or for the vertical (*p* = 0.733) or the diagonal meridian (*p* = 0.163; Bonferroni-corrected *post hoc* comparisons had an adjusted α of 0.017) (Figure 4).

The ANOVA including all valid trials of the modified AWT and of the basic AWT revealed no primary effect for the factor condition, *F*(1, 19) = 0.313, *p* = 0.582, η^2^ = 0.016. However, there was a main effect for the factor meridian, *F*(1.513, 28.753) = 17.327, *p* < 0.001, η^2^ = 0.477, ε = 0.757 (Mauchly’s test of sphericity: χ^2^(2) = 6.985, *p* = 0.030): Bonferroni-corrected follow-up pairwise comparisons showed that the participants’ AW was prolonged along the horizontal rather than the vertical meridian (*p* < 0.001), with no differences between the other comparisons (*p* > 0.05). We found no significant interaction between condition and meridian, *F*(2, 38) = 1.451, *p* = 0.247, η*_*p*_*^2^ = 0.071.

**FIGURE 5:**
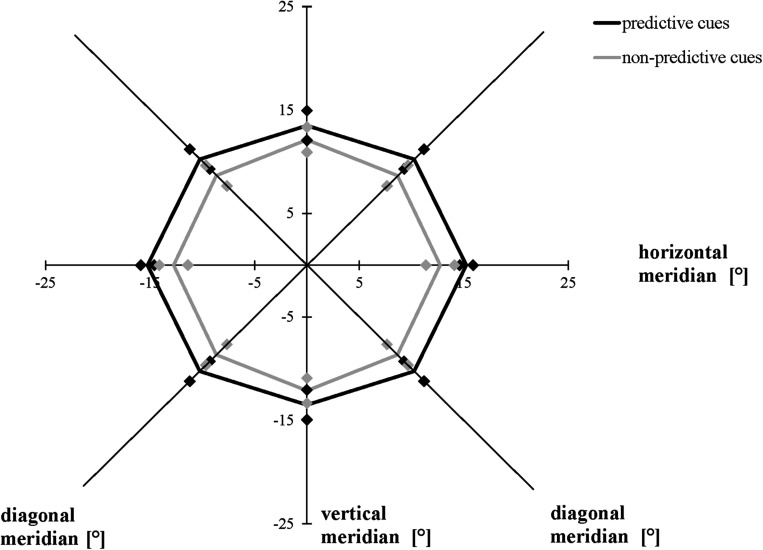
Attention Window with 75% correct performance as a function of meridian and cue predictability in the modified version of the Attention-Window Task with 20% non-predictive and 80% predictive trials. Error bars (indicated by diamond/square symbols) indicate the 95% confidence interval.

The ANOVA including the valid and invalid trial conditions in the modified task revealed that averaging across meridians, participants attained a greater AW when the location of the cues and target stimuli were the same (valid, mean = 28.83°, SD = 4.36°) compared to when they were not the same (invalid, mean = 24.75°, SD = 4.30°), *F*(1, 19) = 9.876, *p* = 0.005, η*_*p*_*^2^ = 0.342. Averaging across conditions, the size of the AW differed as a function of meridian, *F*(2, 38) = 5.543, *p* = 0.008, η*_*p*_*^2^ = 0.226: Bonferroni-corrected follow-up pairwise comparisons showed that the size of the AW was greater for the horizontal rather than the vertical meridian (*p* = 0.028), with no differences between horizontally and diagonally or between diagonally and vertically oriented ones (*p* > 0.05). We did not find a significant interaction between meridian and condition (Figure 5), *F*(1.432, 27.213) = 0.645, *p* = 0.482, ε = 0.716 (Mauchly’s test of sphericity: χ^2^(2) = 9.087, *p* = 0.011).

The results of Experiment 1 confirmed the hypothesis that the use of invalid cue stimuli reduces the spatial elongation of the AW compared to the valid cue condition in the modified task. Participants showed a limitation in their AW by 14% when cue and target stimuli positions were not consistent compared to when the positions were the same. This result is in line with previous research showing performance increases when the target stimuli are presented at locations previously occupied by cues, as opposed to when the target stimuli are presented at new locations (cf. Posner, 1980).

It was initially surprising that participants performed equally well in both tasks (basic AWT, modified AWT), although the modified task included not only valid, but also invalid cues so that the participants were not always aware about the target stimuli positions. This may be explained by the fact that only 20% of all trials in the modified task are non-predictive (invalid). Future research should verify any changes in results if the number of invalid trials were to be increased.

## Experiment 2

Various studies have evidenced an impact of the temporal gap between the presentation of the cue and target stimuli–the SOA–on the reaction to the target stimuli. Müller and Rabbitt (1989), for example, provided a comprehensive investigation of the time course for endogenous and exogenous cues. Previous research has found a facilitation of target detection when SOA is shorter than 200–300 ms (e.g., Posner and Cohen, 1984; Klein, 2000). The facilitation effect has also been detected when the cue predicts an upcoming target location, even at longer SOAs (e.g., Posner, 1980; Egly et al., 1994; Danckert et al., 1998). SOAs being longer than 200–300 ms result in slower response times to cued target locations (Posner and Cohen, 1984; Klein, 2000)–at least when the cues give no predictive information about upcoming target locations. This can be explained by the fact that through longer SOAs the facilitatory effect for the indicated position turns into an “inhibition effect.” This refers to the inhibition of the reorientation of attention to a location that has, shortly before, been looked at already, i.e., a shift of attention from the indicated position to a different one (cf. Kreitz et al., 2020).

By manipulating the SOA between the presentation of the cue and the target stimuli in the AWT, Experiment 2 investigated whether different SOAs (shorter and longer SOAs compared to ones in the basic AWT) affect the maximum extension of the AW. In addition to the basic AWT with an SOA between the peripheral cues and target stimuli of 200 ms, we investigated the effects when the SOA was reduced to 50 ms and when it was prolonged to 350 ms. Based on research showing that peripheral cues produce a stronger facilitatory effect at short rather than at long SOAs (cf. Jonides, 1981), we assumed to measure greater AWs in the 50-ms SOA condition compared to the other conditions with longer SOAs. The peripheral cues indicated the future positions of the target stimuli with 100% validity in all of the three conditions in Experiment 2 and could therefore be defined as predictive cues. Therefore, we expected similar performances for the basic condition (200-ms SOA) and the 300-ms SOA condition as the inhibition effect is typically found for non-predictive cues, but not for predictive cues (cf. Posner, 1980; Egly et al., 1994; Danckert et al., 1998).

### Participants

Twenty participants (11 female and 9 male participants) aged 19 to 34 years (mean_*age*_ = 24.95 years, SD = 4.36 years) took part under the same ethical conditions as in Experiment 1.

### Procedure

To examine the influence of the SOA on the maximum spread and shape of the AW, participants performed three versions of the AWT (each version with a total of 288 trials) in a random order–the basic task with a time interval between the cue and target stimuli of 200 ms, modified version with an SOA of 50 ms, and one with an SOA of 350 ms. They got the same instructions as in Experiment 1, except that they would only see valid pre-cues, and the time between cues and target stimuli could be different.

### Results and Discussion

**FIGURE 6:**
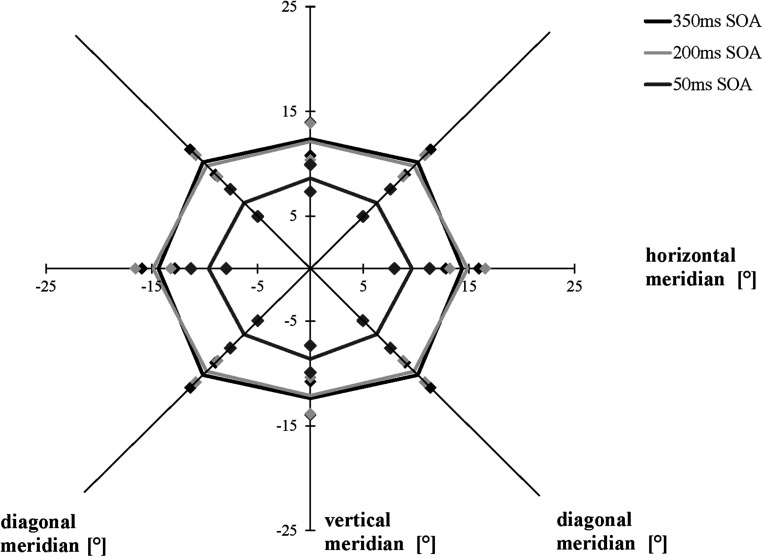
Attention Window with 75% correct performance as a function of meridian and SOA (time interval between the peripheral cues and the target stimuli). Error bars (indicated by diamond/square symbols) indicate the 95% confidence interval.

We compared the separations corresponding to the 75% accuracy threshold of the AW in a 3 × 3 [SOA (50, 200, 350 ms) × meridian (horizontal, vertical, and diagonal)] repeated-measures ANOVA. As in Experiment 1, averaging across the three conditions (50, 200, and 350 ms), the AW varied as a function of meridian, *F*(1.537, 29.194) = 22.131, *p* < 0.001, η*_*p*_*^2^ = 0.538, ε = 0.672 (Mauchly’s test of sphericity: χ^2^(2) = 6.462, *p* = 0.040): Bonferroni-corrected follow-up pairwise comparisons showed that participants had a larger AW along the horizontal meridian compared to the vertical meridian (*p* = 0.010) and the diagonal meridian (*p* = 0.002), with no difference between the diagonal and the vertical meridians (*p* > 0.05), indicating that the elliptical shape of the AW remained constant, averaged across all conditions. The three time conditions (SOAs) led to different performances when averaging across all three meridians, *F*(1.344, 25.540) = 17.022, *p* < 0.001, η*_*p*_*^2^ = 0.473, ε = 0.672 (Mauchly’s test of sphericity: χ^2^(2) = 12.044, *p* = 0.002): Participants showed a larger AW when the time interval between the presentation of the cues and the stimuli was 200 ms than when it was 50 ms (*p* = 0.001), and they showed a larger AW when performing the AWT with a 350-ms SOA compared to a 50-ms SOA (*p* = 0.001), with no difference between the SOA of 350 and 200 ms (*p* > 0.05). The interaction between meridian and time condition was marginally significant, *F*(4, 76) = 2.447, *p* = 0.053 (Figure 6).

As expected, we measured similar maximum expansions of the AW when using the AWT version with a time interval of 350 ms compared to an SOA of 200 ms. This finding confirms previous research that long SOAs (>200–300 ms) and cues carrying predictive information about upcoming target locations evoke facilitation (Klein, 2000). However, contrary to our expectations, the short SOA (50 ms) did not result in a facilitatory effect by increasing the AW; instead, the maximum spread of the AW decreased. In the modified version of the AWT with an SOA of 50 ms, we observed a decrease of the size of the AW by 33% compared to the basic task and the modified version with an SOA of 350 ms. Initially, the performance decrease with a shorter SOA seemed surprising. However, this procedure cannot be compared to most previous research focusing on one peripheral target stimulus and not on the maximum extension of the AW.

There is large variability between tasks as to when facilitation turns into inhibition. Klein (2000), for example, showed that the crossover from facilitation to inhibition depends on the difficulty of the task. Thus, it may be possible that worse performance (i.e., a smaller AW) could be observed if the temporal separation between cue and target is increased (e.g., with an SOA of 500 or 1,000 ms). The effects of such increased SOAs need to be studied in further projects.

In the current experiment, the participants knew that the cues were 100% predictive, and so they could expand or contract their visual focus of attention to these cued locations in order to optimally perceive the target stimuli at the exact location. Probably a longer SOA gave the participants more time to zoom out or to shift their attention to the respective locations in the visual periphery. It might be worthwhile to combine the findings of Experiments 1 and 2 in future research and to use valid and invalid peripheral cues in order to investigate the effects of SOA changes with predictive and non-predictive cues on the AW in more detail. There is evidence that predictive and non-predictive exogenous spatial cues evoke different patterns of behavioral effects in cueing tasks (Vivas et al., 2019): Both cues initially attract attention, but only non-predictive cues provoke inhibitory effects when the time interval between the presentation of the cue and the target stimulus is long enough.

Referring to the findings in Experiment 2, there are more possible explanations. While in the current experiment, the peripheral cues were presented at the same position as the target stimuli, in most studies on exogenous cueing (see Carrasco, 2011), the peripheral cues are usually presented close to but not exactly at the target locations. Therefore, it is possible that the presentation of the cue in the current experiment interfered with target perception because of forward masking or perceptual “blending” of cue and target. This explanatory approach should be picked up in future research, and a modified version of the AWT should be developed presenting the peripheral cues close to but not exactly at the same position as the target stimuli locations.

## Experiment 3

Various studies have confirmed an impairment of cognitive performances when complex objects, in contrast to less complex objects, have to be perceived (Irsik et al., 2016). One possibility to increase the complexity of a visual scene is the increase in the types of elements it contains (Palumbo et al., 2014). Therefore, the aim of Experiment 3 was to investigate performance differences caused by the number of element types in the target stimuli in the AWT. It was assumed that the AW would decrease with increasing stimuli complexity compared to the basic AWT.

### Participants

In Experiment 3, 20 participants (9 female, 11 male) aged 18 to 26 years (mean_*age*_ = 20.85 years, SD = 2.25 years) took part under the same ethical conditions as in Experiments 1 and 2.

### Procedure

**FIGURE 7:**
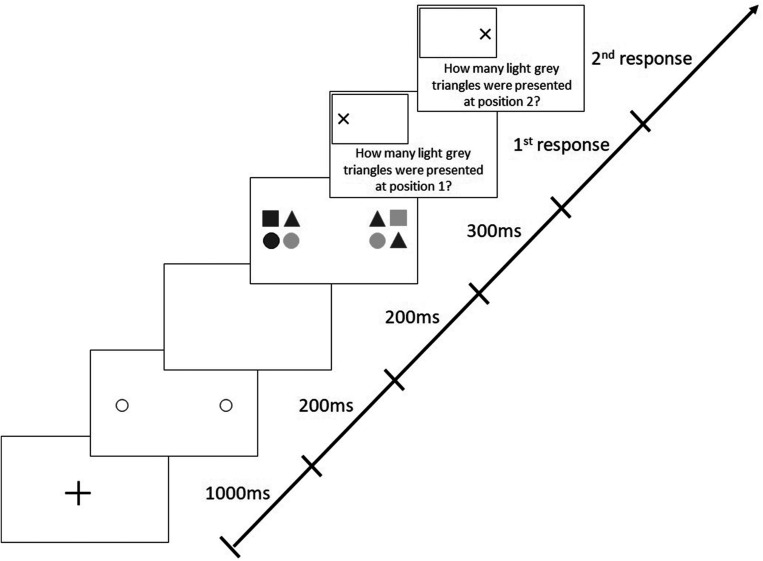
Sequence of events in a trial with target stimuli presentation along the horizontal meridian following valid peripheral cues in the “high complexity” Attention-Window Task.

In the basic version of the AWT, the target stimuli are composed of light and dark gray circles and triangles. Subjects are required to identify the number of light gray triangles in both object formations. In Experiment 3, participants had to perform two versions of the AWT in a random order: the basic task and a modified task with higher complexity. Just as in the basic task, in the high-complexity task, each stimulus group comprised four elements, with the difference that there were six different types of elements to choose from: circles, triangles, and squares of either light or dark gray color (Figure 7). Consequently, there were more possible combinations of composed stimulus groups; the shape (circle, triangle, and square) and shading (light gray, dark gray) of these six different elements varied randomly from trial to trial. The participants’ task was again to identify the number of light gray triangles presented within each stimulus group.

### Results and Discussion

**FIGURE 8:**
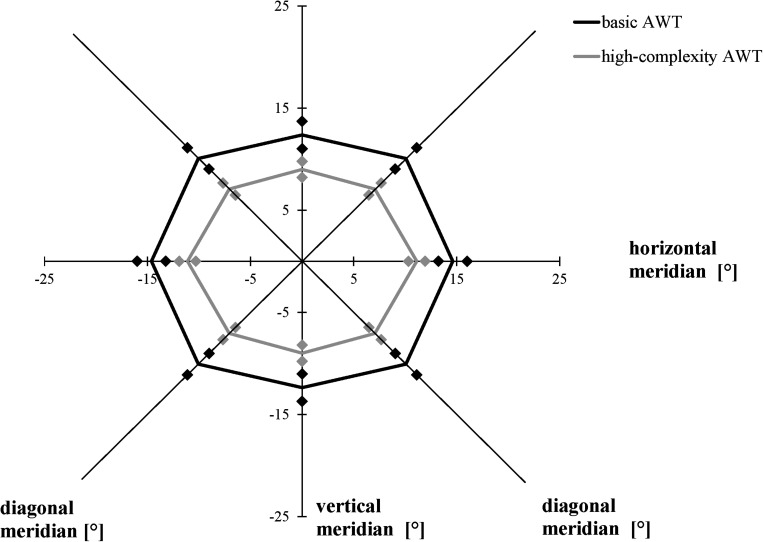
Attention Window with 75% correct performance as a function of meridian and task complexity. Error bars (indicated by diamond/square symbols) indicate the 95% confidence interval.

We performed a 2 × 3 [task (basic AWT, high-complexity task) × meridian (horizontal, vertical, and diagonal)] repeated-measures ANOVA with the AW threshold as the dependent variable. As in Experiments 1 and 2, the size of the AW was defined as the largest stimulus separation where each participant reliably identified the number of light gray triangles in both stimuli on at least 75% of the trials. Averaging across both conditions (basic task, high-complexity task), performance varied as a function of meridian, *F*(2, 38) = 28.575, *p* < 0.001, η*_*p*_*^2^ = 0.601; Bonferroni-corrected follow-up pairwise comparisons showed a wider alignment of the participants’ AW along the horizontal meridian compared to the vertical meridian (*p* < 0.001) and a difference between the vertical and diagonal meridians (*p* < 0.001), with no difference between the horizontal and the diagonal meridians (*p* = 0.057). Averaging across meridians, participants showed larger AWs in the basic task than in the high-complexity task, *F*(1, 19) = 32.303, *p* < 0.001, η*_*p*_*^2^ = 0.630. The interaction between task and meridian was non-significant, *F*(2, 38) = 1.244, *p* = 0.300, η*_*p*_*^2^ = 0.061 (Figure 8).

While object shapes of the target stimuli were limited to triangles and circles in the basic AWT in previous studies so far, the current experiment investigated how far a differentiation of a third possible object shape (square) within a target stimulus influences the size of the AW. In the modified version of the AWT, the results show that it is more difficult to correctly identify the target elements (in this case, the light gray triangles) when they have to be differentiated from more object types (high complexity condition) as in the basic task. We found a decrease in the size of the AW by 29% in this modified version of the AWT compared to the basic task. In both task conditions, the maximum AW was represented in an elliptical shape in line with previous research (cf. Hüttermann and Memmert, 2017).

In all the three experiments, especially in Experiment 3, the participants’ visual short-term memory could have played a role for the attentional performance. This possibility should be explored in future research by using masks after the presentation of the target stimuli. A backward masking could be used to investigate properties of subliminal or unconscious processing (Ansorge, 2003; Vorberg et al., 2003).

## General Discussion

The objective of the current study was to investigate task-specific changes on the maximum spread and shape of the AW by adjustments and modifications of the basic AWT, in order to understand the mechanisms underlying the processing of peripheral stimuli in more detail. While Experiment 1 examined the effects of cue validity on the maximum spread and shape of the AW, Experiment 2 dealt with the effects of the SOA, and Experiment 3 with the effects of target stimuli complexity. The results of Experiment 1 showed that the size of the AW decreased when invalid trials (i.e., different location of cue and target stimuli) compared to valid trials (i.e., same location of cue and target stimuli) were presented in the AWT. By modifying the time interval between cue and target stimuli in Experiment 2, we observed a reduction in the AW with an SOA of 50 ms compared to the standard 200 ms; in contrast, there was no performance difference in the 350-ms SOA condition in comparison with the 200-ms condition. The findings of Experiment 3 confirmed the expectation that the AW would decrease with a higher target complexity (i.e., more types of objects that had to be differentiated). In absolute terms, the greatest restriction (33%) of the AW due to modifications of the AWT across all three experiments was measured when the SOA between the cue and target stimuli was reduced to 50 ms, while we did not find a difference between an SOA of 200 and 350 ms (cf. Experiment 2). The increase of the target stimuli complexity in a modified AWT version led to an AW reduction by 29% compared to the measured performance in the basic AWT (cf. Experiment 3). Non-predictive (invalid) cues limited the maximum AW by 14% in comparison to the trials presenting predictive (valid) cues immediately before the target stimuli appeared (cf. Experiment 1).

In general, there are different options to perceive spatially separated target stimuli at the same time. First, subjects can broaden their focus of attention in a way that these stimuli are all encompassed within one unitary focus following the approaches of the zoom lens model (cf. Eriksen and St. James, 1986). Second, it may be possible that subjects have divided their attention between the two target stimuli (cf. Jefferies et al., 2014). Third, an alternative approach to explain the results of the three experiments–in some ways combining the first two approaches–is the idea that participants were shifting a much narrower unitary window (a unitary beam) between the two target stimuli locations (cf. spotlight model, Posner, 1980). This approach could explain why it takes longer to shift the window when the distance is larger. When the peripheral cues are valid, only a single shift is required from one cued location to the other, whereas with the invalid cues, an additional shift is required. Strictly speaking, we cannot exclude the possibility that the participants shifted spatial attention from one side to the other. It is possible that the participants fixated on the center of the screen while attention was distributed, at first, to one set of stimuli and, afterward, to the second set of stimuli (i.e., periodic sampling). While sampling of attention usually does not take longer than 100 ms (Jans et al., 2010) or 125 ms (Cave et al., 2010; Landau and Fries, 2012), the presentation duration in the AWT was rather long with 300 ms in order to rule out periodic sampling. Future studies should analyze if the current results remain the same when stimuli presentation duration is reduced to less than 100 ms. However, it should also be mentioned that tasks including periodic sampling stimulus material are often less complex than in the AWT (cf. Jans et al., 2010). Subsequently, the AWT would need to be modified to allow a reduction of presentation duration anyway. Furthermore, a mask presentation following each trial in the AWT seems reasonable to explore this approach in more detail in future research. It can be argued that without such a mask it might be difficult to differentiate between a broad AW and a small but rapidly shifting attentional spotlight.

Referring to the results of Experiment 2, 50 ms may possibly be not enough time to shift, which may explain why the AW decreased with the 50-ms SOA between cues and target stimuli, compared to longer SOAs. When the stimuli are more complex, like in Experiment 3, participants possibly need attention for a longer duration in each location, and the shift from the first observed location starts later–resulting in less-than-optimal “attention time” on the second stimulus. Future studies are needed to gain more clarity about the distribution of spatial visual attention within the inner surface of the AW.

While the current study did not investigate any specific differences along the single meridians, e.g., differences between the upper and lower vertical meridian, the findings support previous research showing that the AW was the widest along the horizontal meridian across all tested task versions and conditions. Most previous studies using the AWT have shown that the maximum AW presents itself in the shape of an ellipse with greater horizontal rather than diagonal and vertical orientation (for a review, see Hüttermann and Memmert, 2017). Although the results of Experiment 2 completely and the results of Experiment 1 partly confirm these differences in threshold distance between the meridians, some analyses in Experiment 1 and the data of Experiment 3 showed a significant difference only between the horizontal and vertical meridians, but not between the horizontal and diagonal meridians. Figures 4–6, and 8 presenting the size and shape of the AW, however, visualize that the AW still represents itself in an elliptical shape rather than in a rectangular shape, even though the elliptical shape slightly shifted depending on the task condition.

With reference to the current study, it can be concluded that the AWT, with its possible task changes and adjustments, can be considered a potential standard tool to measure the maximum spread and shape of the spatial AW.

## Data Availability Statement

The raw data supporting the conclusions of this article will be made available by the authors, without undue reservation.

## Ethics Statement

The studies involving human participants were reviewed and approved by German Sport University Cologne. The patients/participants provided their written informed consent to participate in this study.

## Author Contributions

SK and DM developed the study concept and contributed to the design. SK collected the data, analyzed it, and wrote the first draft of the manuscript. Both authors approved the final submitted version of the manuscript.

## Conflict of Interest

The authors declare that the research was conducted in the absence of any commercial or financial relationships that could be construed as a potential conflict of interest.

## Footnotes

1 For analysis purposes, data from the two diagonal meridians was combined resulting in referring to only three meridians in the following.
